# The Evolving Understanding of the Molecular and Therapeutic Landscape of Pancreatic Ductal Adenocarcinoma

**DOI:** 10.3390/diseases6040103

**Published:** 2018-11-13

**Authors:** Ashleigh Parkin, Jennifer Man, Angela Chou, Adnan M Nagrial, Jaswinder Samra, Anthony J Gill, Paul Timpson, Marina Pajic

**Affiliations:** 1 The Kinghorn Cancer Centre, The Garvan Institute of Medical Research, 384 Victoria St, Darlinghurst, Sydney, NSW 2010, Australia; a.parkin@garvan.org.au (A.P.); j.man@garvan.org.au (J.M.); a.chou@garvan.org.au (A.C.); Adnan.Nagrial@health.nsw.gov.au (A.M.N.); Anthony.Gill@health.nsw.gov.au (A.J.G.); p.timpson@garvan.org.au (P.T.); 2 University of Sydney, Sydney, NSW 2006, Australia; 3 Crown Princess Mary Cancer Centre, Westmead Hospital, Sydney, NSW 2145, Australia; 4 Department of Surgery, Royal North Shore Hospital, St Leonards, Sydney, NSW 2065, Australia; jas.samra@bigpond.com; 5 Department of Anatomical Pathology, Royal North Shore Hospital, St Leonards, Sydney, NSW 2065, Australia; 6 Cancer Diagnosis and Pathology Research Group, Kolling Institute of Medical Research, St Leonards, NSW 2065, Australia; 7 St Vincent’s Clinical School, Faculty of Medicine, University of NSW, Sydney, NSW 2010, Australia

**Keywords:** pancreatic cancer, tailored therapy, molecular classification, clinical trials

## Abstract

Pancreatic cancer is the third leading cause of cancer-related deaths, characterised by poor survival, marked molecular heterogeneity and high intrinsic and acquired chemoresistance. Only 10–20% of pancreatic cancer patients present with surgically resectable disease and even then, 80% die within 5 years. Our increasing understanding of the genomic heterogeneity of cancer suggests that the failure of definitive clinical trials to demonstrate efficacy in the majority of cases is likely due to the low proportion of responsive molecular subtypes. As a consequence, novel treatment strategies to approach this disease are urgently needed. Significant developments in the field of precision oncology have led to increasing molecular stratification of cancers into subtypes, where individual cancers are selected for optimal therapy depending on their molecular or genomic fingerprint. This review provides an overview of the current status of clinically used and emerging treatment strategies, and discusses the advances in and the potential for the implementation of precision medicine in this highly lethal malignancy, for which there are currently no curative systemic therapies.

## 1. Introduction

Biomarker directed clinical decision-making to improve treatment outcomes is the contemporary paradigm of cancer care and therapeutic development. However, despite myriad biomarker studies in many cancer types, few have been validated and implemented in clinical practice [1]. The central challenge is matching molecular phenotypes with their correct clinical phenotype (therapeutic responsiveness). The heterogeneity of response even in the case of established biomarkers, and unknown frequencies of responders in biomarker negative populations, together with multiple molecular phenotypes conferring response to specific agents (for example, PARP inhibitor responsiveness in tumours with BRCA1, BRCA2, PALB2, and PTEN aberrations) generates significant complexities and challenges in advancing these therapeutic strategies.

Pancreatic ductal adenocarcinoma (PDAC), which constitutes 90% of all pancreatic cancers, is currently the third leading cause of all cancer-related deaths and will become the second by 2030 [2], presenting a major health issue in the community. This is a highly lethal malignancy, with an overall 5-year survival rate of only 8% [2,3]. This extremely poor outcome is partly due to the majority of the cases being diagnosed when pancreatic cancer has already spread to distant sites, with 5-year survival rates in metastatic disease being only 3% [3].

Moreover, pancreatic cancer appears to be particularly heterogeneous, and apart from a few notable exceptions, which have not been successfully targeted, most genetic aberrations occur at a frequency of <5% [4,5,6]. Hence, even if these mutations are effectively targeted, it is unlikely that an overall benefit would be detected with non-targeted population clinical trial designs, unless combinations are used (for example, combination of chemotherapeutics fluorouracil, leucovorin, irinotecan and oxaliplatin or FOLFIRINOX [7]. Current standard therapies for patients with advanced PC, in the form of FOLFIRINOX or Gemcitabine and nanoparticle albumin-bound paclitaxel (Abraxane), have shown a significant but modest clinical benefit and a marginal survival advantage of just 4.3 and 1.8 months, respectively, in unselected populations [7,8].

The central model of PDAC development involves gradual increasing mutational burden, with consecutive alterations in *KRAS*, *CDKN2A*, *TP53* and *SMAD4* genes contributing to accelerated progression from early pancreatic intraepithelial neoplastic lesions (PanIN) to late-stage metastatic disease [9,10,11]. However, due to the highly aggressive and metastatic clinico-pathological behaviour of pancreatic tumours, and the thus far ineffective considerable efforts aimed at early detection all support the notion that pancreatic cancer progression is not a gradual process [12]. In fact, recent comprehensive analyses of >100 whole genomes from purified primary and metastatic pancreatic tumours suggest that “punctuated equilibrium”, where pancreatic cancer development can be divided into two major events: the early cancer-initiating event and subsequent, rapid cancer-transforming event, may more appropriately reflect the clinical PDAC disease progression [13]. Specifically, according to this model, most mutations would accrue in an extended phase of preneoplastic tumour development, not necessarily in a linear fashion. Cancer transformation, likely resulting from increased genomic instability due to a “catastrophic” event, such as chromothrypsis [14], would then lead to generation of invasive clones, with subsequent rapid dissemination and colonisation of distant sites.

Adding to the genomic complexity, the desmoplastic stroma that envelops pancreatic cancer cells in growing tumours, not only presents a physical barrier to therapeutic efficacy, but at the same time, presents an environment that actively produces pro-tumourigenic, immunosuppressive signals that further drive pancreatic tumourigenesis, disease progression and treatment resistance [15,16]. New strategies that involve design of tailored treatments and combinations that target different components of a developing tumour, in smaller, well-defined subgroups of patients are sorely needed. This review provides an analysis of the diverse molecular characteristics of PC, challenges with the current treatment landscape of metastatic disease, and presents the latest advances in therapeutic targeting, with a particular focus on the potential of precision medicine strategies for pancreatic cancer.

## 2. Clinical Presentation

PDAC is a notoriously insidious cancer, frequently presenting with vague, non-specific symptoms that are commonly observed for multiple abdomen or gastrointestinal tract pathologies. The classic presentation is with the triad of epigastric abdominal pain, weight loss and jaundice, which rapidly worsen as disease progresses and lead to a substantial deterioration in quality of life [17]. However, presentation of symptoms varies according to the location of the tumour within the pancreas. Tumours in the head of the pancreas more commonly present with jaundice, steatorrhoea and weight loss [18], with back pain associated with tumours originating in the tail of the pancreas [19]. Adult onset diabetes mellitus presents both an early manifestation and an etiologic factor of PDAC [20]. In metastatic disease, additional symptoms can include an abdominal mass, ascites, lymphadenopathy and bone pain. Diagnosis of PC is performed using a combination of established methodologies involving initially abdominal ultrasonography, followed by more advanced techniques, such as computed tomography and magnetic resonance imaging in combination with endoscopic ultrasonography [21]. Use of invasive methods is necessary to accurately diagnose PDAC, without which there are significant difficulties in differentiating between malignant disease, benign pancreatic lesions, or chronic pancreatitis. Of note, development and future validation of novel blood-based biomarkers that detect somatic mutations or “liquid biopsies” [22,23], or circulating exosomal biomarkers [24,25] may present promising new options and a minimally invasive alternative to direct tumour biopsy.

### 2.1. Clinicopathological Staging of PDAC

After a definitive diagnosis of PDAC, clinical staging is utilised to determine the optimal treatment approach for the patient. The American Joint Committee on Cancer TNM staging system is widely utilised worldwide as the most authorised tool for tumour staging assessment. Staging assessment is based on the extent of invasion into the pancreas and surrounding tissue (T), presence or absence of spread to lymph nodes (N), and presence or absence of metastasis (M). In October of 2016, AJCC/UICC released the 8th edition, which incorporated significant changes in the T and N classification of PDAC. In the 8th edition, stages T1–T3 are redefined specifically based on tumour size (T1 ≤ 2 cm; 2 cm ≥ T2 ≤ 4 cm; T3 > 4 cm). When the tumour invades the celiac axis, common hepatic artery and/or superior mesenteric artery, it is defined as T4, with the classification as “unresectable” (from AJCC 7th edition 2010) now removed. The N classification was further subdivided according to the number of positive lymph nodes as N0, N1 (≥1 and ≤3) and N2 (>3). In the 8th edition, T1–3N2M0 was defined as stage III, and the other stages, including Stage IV (metastasised tumours), remain unchanged. Importantly, the system provides useful stratification of patient survival and resectability by stage [26,27].

### 2.2. Current Treatment Approaches for Advanced PDAC

Unfortunately, most patients are routinely diagnosed with already advanced, metastatic disease. Gemcitabine monotherapy was established as standard of care treatment for PDAC in 1997 [28], demonstrating superior response rate over 5-fluorouracil. Lack of subsequent further dramatic improvement in patient outcomes is certainly not due to lack of trying. Multiple phase II and III studies have attempted to improve upon gemcitabine efficacy either by modulating its pharmacokinetics [29] or by combining with other agents [30,31,32].

More recently, two different chemotherapeutic combinations (FOLFIRINOX or gemcitabine plus Abraxane) have been shown to significantly improve survival in advanced disease in well patients (FOLFIRINOX: median overall survival 11.1 vs. 6.8 months for gemcitabine monotherapy, *p* < 0.001; gemcitabine and Abraxane: median overall survival 8.5 vs. 6.7 months for gemcitabine, *p* < 0.001) [7,8], with recent analyses suggesting comparable real world efficacy [33]. FOLFIRINOX or gemcitabine and Abraxane are both utilised as first-line agents in metastatic PDAC and are administered to well patients, with gemcitabine monotherapy, as a more tolerable treatment, still prescribed for the elderly or patients with a poor performance status [34]. Recent update to the treatment guidelines also recommends immunotherapy pembrolizumab for patients who fail 1st line therapy and whose tumours harbour mismatch repair deficiency/microsatellite instability [35].

New treatment combinations are also on the horizon, with recent data from the Phase IIb PACT-19 trial suggesting that a combination of cisplatin, Abraxane, capecitabine, and gemcitabine increased progression-free survival compared with gemcitabine and Abraxane in the metastatic setting [36]. Validated predictive biomarkers of treatment response to these combinations are currently lacking and needed, to further improve identification of patient subgroups most likely to respond to each regimen.

## 3. The “omic” Diversity of PDAC

Since the first genomic analysis of PDAC in 2008 [37], with exponential advances in sequencing methodologies and associated bioinformatics approaches, PDAC has been gnomically and transcriptomically characterised to an unprecedented depth [4,5,38,39]. Early studies identified the 12 key pathways and processes whose component genes were genetically altered in most pancreatic cancers, including K-Ras, transforming growth factor β (TGFβ), c-Jun N-terminal kinase, integrin, Wnt/Notch and Hedgehog networks, small GTPase-dependent signalling, G1/S cell cycle checkpoint regulation, invasion, homophilic cell adhesion, apoptosis and DNA repair pathways [37]. In 2011, using gene expression microarray profiling of resected PDAC specimens, three subtypes of pancreatic cancer were defined: “quasi-mesenchymal”, which was associated with poor prognosis, “classical”, and “exocrine-like”. These subtypes were found to have differential response to therapeutic agents, with pancreatic cancer cell lines that were of “classical” subtype displaying resistance to gemcitabine, but sensitivity to erlotinib in vitro. In contrast, the “quasi-mesenchymal” lines were inversely gemcitabine-sensitive, but erlotinib-resistant [39]. Thus far, these predictive signatures of treatment response have not been translated into clinical application.

In 2015, a comprehensive whole genome sequencing (WGS) analysis of 100 primary operable PDAC cases further stratified pancreatic cancer into four major subtypes based on the extent of structural variation (SV) [5]. PDAC was stratified into:

(1) “stable” subtype, present in 20% of all patients whose tumour genomes harboured fewer than 50 SV events;
(2) “locally rearranged” subtype, detected in 30% of the cohort, characterised by a single focal event on one-two chromosomes, breakage-fusion-bridge events, chromothripsis or low prevalence alterations in known oncogenes and therapeutic targets (focal amplifications in *KRAS*, *SOX9*, *GATA6*, *ERBB2*, *MET*, *CDK6*);
(3) the “scattered” subtype, present in 36% of tumours, showed a range of non-random chromosomal damage with less than 200 structural rearrangements;
(4) the “unstable” or high SV subtype, present in 14% of PDAC, characterised by a large extent of SV (>200 events), suggesting major defects in DNA maintenance, with associated increased sensitivity to DNA-damaging agents [5]. Deleterious mutations in *BRCA1*, *BRCA2* and *PALB2* genes, essential components of homologous recombination-mediated DNA repair, were associated with the “unstable” PDAC subtype, and similarly, the top quintile of the previously identified BRCA mutational signature [40], was present in the majority (10/14) of unstable genomes [5].

Further comprehensive integrated genomic/transcriptomic analysis of 456 pancreatic cancers [4], defined four PDAC subtypes, based on the differential expression of transcription factors and downstream targets critical during pancreas development, differentiation and regeneration [4]. PDAC was classified into:

(1) “squamous” subtype, associated with the worst patient prognosis;
(2) “pancreatic progenitor” subtype, enriched for transcriptional networks containing *PDX1*, *MNX1*, *HNF4G*, *HNF4A*, *HNF1B*, *HNF1A*, *FOXA2*, *FOXA3* and *HES1* genes;
(3) “aberrantly differentiated endocrine exocrine” (ADEX) subtype, a sub-class of the “pancreatic progenitor” group, defined by transcriptional networks that are essential in later stages of pancreatic development and differentiation. These include upregulation of *NR5A2*, *MIST1*, *RBPJL* and their downstream targets, which regulate acinar cell differentiation and pancreatitis/regeneration;
(4) “immunogenic” subtype, associated with a significant immune infiltrate, with predominant expression profiles related to infiltrating B and T cells, upregulation of *CTLA4* and *PD1* immuno-suppressive pathways, inferring therapeutic opportunities with immune modulating agents for specific tumours in this class.

**Figure 1:**
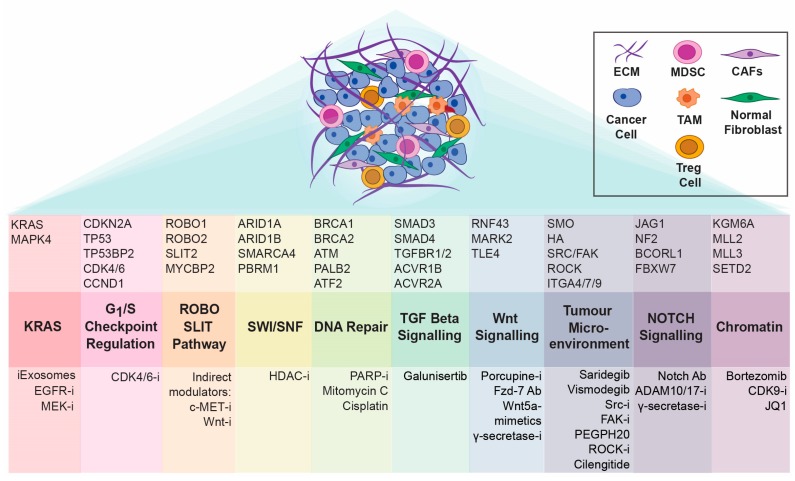
Frequently altered signalling pathways that drive pancreatic cancer progression, adapted and modified based on [4,37]. Key aberrations of interest, and associated targeted therapies in pre-clinical/clinical development, including small molecule inhibitors (i), antibodies (Ab) and other agents of interest are depicted. ECM: extracellular matrix; TAM: tumour-associated macrophage; MDSC: myeloid-derived suppressor cell; T-reg: regulatory T cell; CAF: cancer-associated fibroblast.

Building on these findings, Connor et al. [41] subsequently provided further insight into the molecular pathology of PDAC, describing an interesting correlation between signatures that define double-stranded DNA break repair (DSBR) and mismatch repair (MMR) deficiencies and specific immune profiles. Specifically, genes associated with increased cytolytic activity of infiltrating CD8-positive T lymphocytes plus increased expression of immune checkpoint genes (CTLA-4, PD-L1, PD-L2, and indolamine 2,3-dioxygenase 1 (IDO-1) were increased in DSBR and MMR cases within the examined cohort [41], which was similar to the expression patterns observed in melanomas responsive to checkpoint blockade. Importantly, this suggests that similar to other solid cancers [1], pancreatic tumours with a high mutation burden may present a viable target for immune-modulating combination therapies. Despite the explosion in “omic”-characterisation of pancreatic cancer, which has produced unprecedented insights into the complex mutational and changing landscape of this disease, the established molecular taxonomy is yet to be utilised clinically when establishing effective treatment plans. Moreover, several networks, including ROBO/SLIT and TGF-beta signalling, can play either a tumour-suppressive or tumour-promoting role, that is highly context and cell type dependent, and are therefore difficult to target therapeutically [42,43,44,45,46]. This duality assigned to TGF-beta function, for example, was recently comprehensively examined in select genetic models of PDAC, whereby the authors showed that the TGF-beta tumour suppressive role involves epithelial-to-mesenchymal transition (EMT)-associated disruption of a key oncogenic transcriptional network [44]. This is particularly interesting, given that EMT, a developmental program previously associated with acquisition of invasive, highly malignant features in pancreatic tumours [47], here through a Sox4-dependent mechanism was shown to promote apoptosis [44]. Despite these challenges, characterisation of several major pathways frequently altered in PDAC [4,37] has already identified numerous opportunities for therapeutic development, [42,48,49,50] (Figure 1), with early successes already on the horizon [50,51,52], and discussed below.

### 3.1. Targeting KRAS

Mutationally activated KRAS (Kirsten rat sarcoma viral oncogene homolog) is the most frequently occurring alteration in PDAC (94% of cases; [5,6,37]) and a major contributor to therapeutic resistance. Sustained, aberrant activation of KRAS that leads to uncontrolled cell proliferation is largely driven by point mutations at residues G12, G13 and Q61. Moreover, activation of distinct downstream signalling cascades has been linked to specific KRAS mutants, where KRASG12D predominantly leads to activation of MAPK and PI3K pathways, whereas KRASG12V activates Ral signalling [53], thus potentially influencing the response to KRAS-driven treatment strategies. The main past and current strategies for developing therapeutics to block mutant KRAS function have thus far been largely disappointing [54]. With RAS GTPases requiring farnesylation, an essential lipid post-translational modification required for their malignant transforming activity [55], development of farnesyl transferase inhibitors presented a logical next step. Unfortunately, the early promising preclinical findings with these compounds did not translate into meaningful clinical benefit [56,57]. Of note, recent developments utilising exosomes that were derived from normal mesenchymal cells and packed with short interfering RNA specific to *KrasG12D* oncogene (iExosomes), suggest that this may present a powerful new approach for targeting Kras-mutant PDAC. Specifically, Kamerkar et al. 58 identified presence of CD47 integrin on exosomes and Ras-induced macropinocytosis as two main mechanisms by which exosome clearance from circulation can be effectively reduced, at the same time enhancing specific targeting and improving delivery of loaded siRNA/shRNA targeting KrasG12D to pancreatic cancer cells [58]. Moreover, authors demonstrated potent single agent in vivo activity in multiple human orthotopic and genetically engineered mouse models of Kras mutant PDAC. Clinical-grade iExosomes have recently been produced [52], with a Phase I dose-finding and tolerability study already underway (NCT03608631). In parallel, targeting of KRAS effector signalling holds significant promise for clinical translation and has become the focus of numerous studies [59,60,61], with comprehensive recent reviews on this topic [45,60,62].

Conversely, distinct therapeutic options may be available for patients whose pancreatic tumours harbour wild-type KRAS. Treatment of advanced PDAC with a combination of gemcitabine and epidermal growth factor receptor (EGFR) inhibitor erlotinib, has led to a marginal therapeutic benefit (median survival of 6.24 months compared with 5.91 months for gemcitabine alone) [63], which lost significance when this combination was examined in all-comers in the adjuvant setting [64]. Further data analyses from trials investigating EGFR inhibitor efficacy, revealed that patients who developed skin rash during erlotinib treatment (an established adverse effect of drugs that target EGFR signalling) had considerably improved prognosis with 1-year survival rates beyond 40%, comparable with previous reports for FOLFIRINOX [63,65,66,67]. Moreover, the improvement in survival following combined Gemcitabine/EGFR inhibition therapy has also specifically been associated with KRAS wild-type tumour status [66,68,69], suggesting that this combination may be of considerable benefit in a small, but potentially well-defined subgroup of patients with PDAC.

### 3.2. G_1_/S Checkpoint as a Therapeutic Target in PDAC

Cell cycle checkpoints represent essential control mechanisms in healthy cells that ensure accurate cell division. Apart from p53, the p16-cyclin D-CDK4/6-retinoblastoma protein pathway (CDK4 pathway) is another critical control that promotes the G1/S-phase cell cycle transition. Under physiological conditions, Cyclin D complexes with its catalytic partners, cyclin-dependent kinases (CDKs) 4 and 6, driving retinoblastoma protein (RB) phosphorylation and G1 phase progression [70]. Of note, the CDK4 pathway is frequently deregulated in several cancers, including PDAC [4,37], with the p16INK4A tumour suppressor inactivated in 80–90% of clinical specimens [4,71].

Early evidence indicating Cyclin D/CDK4 as an oncogene has stimulated research into the development of small-molecule CDK inhibitors as cancer therapeutics. Pan-CDK inhibitors have shown limited efficacy in clinical trials, however selective CDK4/6 inhibitors, such as PD-0332991 (palbociclib) or LY2835219 (abemaciclib), have emerged as a powerful class of agents with clinical activity in a number of malignancies, firstly demonstrated in the treatment of ER+/HER2- metastatic breast cancer [72,73], and other solid cancers [74]. As a high proportion of pancreatic tumours carry aberrations in G1/S checkpoint machinery, targeting PDAC subtypes that are dependent on CDK4/6 signalling may therefore be a reasonable therapeutic approach. Combinations involving dual CDK4/6 and mTOR targeting have shown promise in preclinical studies [75,76] and have been translated into a clinical non-biomarker driven trial (NCT02981342; [77]), however, after the recruitment of the first 80 patients, this specific study has been terminated (awaiting publication of results).

Comprehensive preclinical exploration of the long-term responsiveness to CDK4/6 inhibition and CDK4/6 inhibitor-based combinations, highlights the need for a more personalised approach in the treatment of pancreatic cancer, with RB as a potential companion biomarker that may help enrich for responders to CDK4/6 inhibitor-regimens [78]. Moreover, recent studies in PDAC and other cancers, suggest a considerably more complex mechanism of action for CDK4/6 inhibitors that includes multifaceted global inhibitory effects on tumour cells, stromal cells and extracellular matrix (ECM) organisation at different stages of PDAC progression [78], inhibition of epithelial-to-mesenchymal transition (EMT) signalling in breast cancer metastasis [79] and improved anti-tumour immunity by enhancing T-cell activation [80,81]. These new features need to be incorporated into future trial design, and indeed several trials are including a retrospective or prospective analysis of potential companion biomarkers (including tumour RB expression by immunohistochemistry plus *CDK4/6* amplification or *CCND1* amplification) as part of the clinical assessment of PD-0332991 efficacy in PDAC (The MATCH Screening Trial NCT02465060, NCT02501902).

### 3.3. Targeting DNA Damage Repair Signalling in PDAC

Maintenance of cellular genomic integrity is regulated by a complex network of DNA damage response (DDR) proteins, which are readily activated by endogenous and exogenous mitogens, including reactive oxygen species and cytotoxic agents. Importantly, deregulation of this highly organised network, detected in approximately 9–14% of human PDAC 4,5 could be therapeutically exploited [82].

*BRCA1* and *BRCA2* are well-characterised tumour suppressor genes that when heterozygously mutated in the germ line, increase the risk substantially for several malignancies, including breast, ovarian, pancreatic and prostate cancer [83,84]. Functional BRCA1 and BRCA2 proteins are essential for the repair of genotoxic double-stranded DNA breaks through a high-fidelity pathway called homologous recombination (HR) [82]. Cancers that arise in individuals with a germline mutation in *BRCA1/2*, frequently acquire a somatic loss-of-function aberration in the corresponding wild-type *BRCA* allele, leading to HR repair deficiency. In addition to BRCA1 and 2 mutations, aberrations in other genes (incl *PALB2* [85,86], BRCAness [40] and/or high extent of structural rearrangement [5], may lead to loss of functional HR, and importantly, may sensitise these cancers to specific DNA-damaging treatments, including poly(ADP-ribose) polymerase (PARP) inhibitors and DNA-intercalating agents (mitomycin C, platinum-based combinations).

Platinum agents have been previously combined with gemcitabine and examined clinically in “all-comers”. Although findings from single trials [87,88] suggest only trending (but not statistically significant) improvements in overall survival following combination treatment, a pooled analysis of two international multi-centre trials suggests that combining gemcitabine with a platinum analogue may be of significant therapeutic benefit in advanced pancreatic cancer (HR = 0.81; *p* = 0.031 [89]), and may be further improved upon by adding a companion biomarker. In fact, selected early case studies have already highlighted the potential of personalising these treatment strategies in PDAC. Specifically, addition of cisplatin after progression on gemcitabine monotherapy, led to a complete clinical response in a patient with a pathogenic germline *BRCA2* (1153 insertionT) mutation [90]. Similarly, a patient with a PDAC tumour harbouring biallelic inactivation of the *PALB2* gene, had an exceptional response to mitomycin C [86]. Furthermore, a review on the impact of *BRCA1* and *BRCA2* germline mutations and therapeutic outcome observed superior overall survival in advanced *BRCA*-associated PDAC with platinum exposure (*n* = 71 patient study) [91]. Subsequent Phase II trial involving PDAC patients with germline *BRCA* mutations has since confirmed that PARP-inhibitor olaparib offers a clinical benefit [92], with similar effects observed with rucaparib in a study of 19 pre-treated patients with *BRCA*-mutant cancer [93], although another PARP-inhibitor veliparib did not elicit a significant response in this setting [94]. Of note, the PARP catalytic inhibitory activities of various PARP inhibitors in clinical testing do not correlate strongly with respect to cytotoxic and trapping potency; for example, olaparib has shown greater cytotoxic and PARP-trapping activity than veliparib in vitro [95] and this could potentially infer differences in clinical potency between the various PARP-targeting agents.

With growing evidence supporting the clinical development of PARP- or platinum-based regimens (including FOLFIRINOX [85]) in the treatment of *BRCA*-mutated PDAC, the National Comprehensive Cancer Network (NCCN) has recommended consideration of a first-line platinum-based regimen in patients with advanced PDAC and a hereditary cancer syndrome involving a DNA repair mutation [96]. Ongoing clinical studies further aim to assess the tolerability and efficacy of PARP-inhibitor based combinations regimens (NCT01585805), or their utility as maintenance monotherapy after first-line platinum-based chemotherapy in *BRCA1*, *BRCA2* or *PALB2*-mutant pancreatic cancer (NCT02184195, NCT03140670).

### 3.4. Mismatch Repair Deficiency in PDAC

During the malignant transformation process, pancreatic cancer cells acquire multiple mechanisms to evade the immune response. Antibodies that target these inhibitory signals called immune checkpoint inhibitors, although highly successful in the treatment of certain solid cancers [97,98], have thus far not demonstrated significant activity in PDAC, when examined without a companion biomarker [99,100,101]. More recently, deficiency in mismatch repair has been effectively utilised to predict response to immunotherapy agents in the treatment of metastatic colorectal and other cancers [102]. Mismatch repair (MMR) is another highly conserved mechanism for the repair of DNA lesions, which recognises and repairs small loops within the duplex DNA that arise from nucleotide misincorporation, either by base–base mismatches or by insertion/deletion loops [103]. Defects in MMR lead to genome-wide instability, particularly in simple repetitive sequences, known as microsatellite instability.

MMR deficiency is rare in PDAC and accounts for approximately 1% of cases [4], however as these patients present with a significantly higher burden of mutations that may lead to higher immunogenicity, there is strong rationale for the use of checkpoint inhibitors in this setting. The recently published, expanded Phase II study by Le et al. [1] demonstrated that significant responses to immune checkpoint inhibitor pembrolizumab were observed only in patients with MMR-deficient tumours. A pancreatic cancer-focussed study revealed that 57% of the MMR-deficient patients (7/833, 0.8% frequency of MMR-deficiency) treated with immune checkpoint blockade had treatment benefit (one complete response, two partial responses, one stable disease) [104]. These promising results have since led to the first ever cancer-agnostic FDA approval of pembrolizumab for biomarker-defined disease, (MMR-deficient malignancies, including PDAC) [50].

## 4. Tumour Microenvironment Matters: Exploration of Stromal Components as Therapeutic Targets in Pancreatic Cancer

The pancreatic tumour micro-environment (TME) comprises both cellular elements and marked desmoplasia, that collectively not only form an effective physical barrier leading to limited drug penetration, but also through dynamic cancer cell-stromal cell crosstalk, directly promote cancer growth, survival and treatment failure [105]. The cellular components within the TME are incredibly diverse, and include myofibroblast, immune, neuronal, adipose cells, the blood and lymphatic vascular networks, all of which are embedded in a dense ECM, rich in hyaluronic acid (HA), collagens, fibronectin, laminin and proteoglycans [105]. As TME plays an important role in PDAC progression, it is therefore not surprising that it also presents an attractive therapeutic target, and a highly active area of investigation.

Early promising treatment strategies designed to deplete tumour-associated stromal tissue by targeting the Hedgehog pathway, did not however, translate into a successful clinical approach. Olive et al. [106] were the first to show that co-administration of Hedgehog signalling inhibitor, saridegib, with gemcitabine produced a transient increase in intra-tumoral vascular density and intra-tumoral levels of gemcitabine, leading to improved survival in an aggressive, genetically engineered Kras^G12D/+^;p53^R172H/+^;Pdx-1-Cre model of PDAC (KPC model [107]). When this approach was tested in humans, however, the opposite occurred [108] and moreover, associated data from the examination of additional pre-clinical models of PDAC (Kras^G12D^; p16/p19^fl/fl^; Pdx1-Cre and Kras^G12D^; p53^R270H/wt^; Pdx1-Cre) revealed concordance with the measured lack of clinical response to Hedgehog inhibition [108]. Subsequent studies have revealed new insight into the potentially diverse roles of the stroma, whereby at least some stromal constituents, driven through Hedgehog pathway signalling, can act to restrain rather than promote tumourigenesis and PDAC progression [109,110].

Modulation of ECM mechanics and cytoskeleton stability occurs through signalling via a complex network that includes integrins, the focal adhesion kinase (FAK)/proto-oncogene tyrosine-protein kinase SRC activation and downstream stimulation of Akt/PI 3-kinase and Rho/ROCK pathways [111,112]. Increased deposition and cross-linking of ECM proteins provides both traction required for tumour cell movement and invasion, and can simultaneously mechanically activate pro-survival and pro-tumourigenic signalling in pancreatic cancer cells that further drives metastasis and disease progression [113,114]. Numerous inhibitors have been developed that target specific components of these complex networks [49,115,116,117,118], some of which are particularly promising, and are highlighted below.

The FAK non-receptor tyrosine kinase is a multifaceted regulator of cell signalling within the TME, and is frequently overexpressed and activated in various advanced-stage solid cancers including PDAC [119,120]. Recent findings indicate that targeting FAK signalling may be of significant therapeutic benefit in PDAC [15,119,121]. In addition to significant anti-proliferative activity in 2D and 3D in vitro pancreatic cancer cultures [119,121], FAK inhibition significantly inhibited pancreatic tumour progression in vivo in the KPC model of PDAC [15], and extended the anti-tumour response to gemcitabine and Abraxane combination in patient-derived PDAC xenograft models 121. Mechanistically, FAK inhibitor treatment further reduced the fibrotic reaction in pancreatic tumours and decreased numbers of infiltrating tumour-promoting myeloid-derived suppressor cells, tumour-associated macrophages and regulatory T-cells, sensitising the otherwise resistant KPC tumours to immune checkpoint inhibition [15]. These interesting pre-clinical findings have now been translated into considerable clinical trials activity, with several randomised trials underway to examine the potential of combining FAK inhibitors (defactinib, GSK2256098) with immunotherapy and/or chemotherapeutic regimens (NCT02758587, NCT02546531, NCT02428270). Interestingly, FAK inhibitor monotherapy has already shown significant clinical activity in other solid cancers, with pronounced efficacy in cancers that harbour loss of specific tumour suppressive signals, such as merlin (encoded by *NF*2 gene), both in preclinical models [122] and clinical studies [123]. Although heterozygous losses or inactivating mutations at the *NF*2 locus occur in approximately 10% of human PDAC [4,41], merlin expression is lost in >40% of PDAC, and is negatively correlated with tumour stage, regional lymph node metastasis and differentiation [124]. We envisage that future trials may incorporate merlin loss and other potential companion biomarkers, to further optimise patient selection and identification of clinical responders to FAK-inhibitor based treatment strategies.

Small molecule inhibitors that target Rho GTPase or its downstream signalling including Rho-associated kinases (ROCK), have also demonstrated significant anti-tumour activity in various pre-clinical models of PDAC [125,126,127] and other cancers. Of note, short-term inhibition of ROCK activity, via oral administration of small molecule inhibitor, fasudil, as a priming agent before administration of standard therapy, gemcitabine, reduced fibrosis, improved tissue perfusion in pancreatic tumours and significantly improved survival in pre-clinical models of pancreatic cancer [127]. Fasudil is an off-patent agent used clinically in the management of stroke and other vascular disorders [128,129], and may present an attractive candidate for drug repurposing as an anti-cancer therapy. Alternatively, novel ROCK inhibitors, including Ripasudil or AT13148 [126], may present another viable treatment option, particularly when applied using a short-term or “priming” regimen, which may enable more effective design of multi-targeted treatment combinations, minimising toxicity frequently associated with chronic drug administration. As the observed anti-tumour activity with ROCK inhibitor “priming” was particularly prominent in pancreatic tumours that were characterised by a high “ECM signature” and stromal remodelling [127], we envisage that these therapies will be of most benefit when coupled with a companion biomarker. Further research is underway to help define clinically useful biomarkers of treatment response to ROCK-targeting.

Another major therapeutic advance involves the development of agents that break down a key ECM component, hyaluronic acid (HA). HA is a large glycosaminoglycan, which raises the interstitial gel fluid pressure within tumours and effectively reduces drug delivery to malignant cells [130]. Given its abundance within the PDAC TME, the efficacy of agents that effectively break down HA, such as pegylated recombinant human hyaluronidase (PEGPH20), has been extensively studied in this setting. Initial studies have demonstrated promising preclinical activity of PEGPH20 in the KPC model of PDAC, with HA degradation leading to normalisation of interstitial fluid pressures and re-expansion of the microvasculature by increasing the diameter but not the total number of blood vessels within PDAC tumours [131,132]. When combined with standard therapy, gemcitabine, PEGPH20 treatment caused a near doubling of overall survival in this highly aggressive autochthonous pancreatic cancer model [131,132]. Clinical evaluation of PEGPH20 efficacy is well underway, with Phase II data already demonstrating significant efficacy of this agent when combined with chemotherapy, effect particularly prominent in patients with HA-high tumours [51]. Levels of intra-tumoural HA have been explored as a predictive biomarker, and can be quantified by immunohistochemistry [133].

The strong preclinical and clinical evidence for HA-targeting in PDAC has led to several ongoing Phase II/III studies which aim to examine the therapeutic potential of PEGPH20, in combination with standard chemotherapies (NCT01839487, NCT02487277, NCT02715804), or immune checkpoint inhibitors (NCT03481920; NCT03634332), in HA-high molecular subgroups of pancreatic cancer. Collectively, these studies highlight stromal signalling as a valid target for the development of precision medicine strategies in PDAC as well as other cancers, with development of additional predictive biomarkers warranted, in the hope of further improving the selection of patients likely to derive the most clinical benefit from these types of therapies.

## 5. Concluding Remarks

Despite several decades of experience with numerous chemotherapies and combinations, pancreatic cancer remains a highly resistant malignancy, with no curative systemic treatments. As our understanding of the complex molecular landscape of PDAC continues to improve, there is a clear need for a fundamental shift in clinical oncology to utilise molecular taxonomy, where individual cancers are selected for optimal therapy depending on their molecular subtype. Moreover, deeper characterisation of the intricate and dynamic cross-talk between the diverse tumour cell types and associated cell signalling pathways will be critical for improved design of novel treatment approaches.

Although not yet standard practice, as highlighted in this review, new clinical studies are increasingly implementing a precision medicine approach as part of trial design, and the results of these studies are eagerly awaited. Further development of multi-agent combinations, necessary in the treatment of PDAC, will also likely benefit from the development of more innovative dosing regimens that may employ (a) biologically effective dose vs. maximal tolerable dose of targeted agents and/or (b) short-term or “priming” treatment strategies, sufficient to ensure maximal efficacy in terms of effective target engagement/modulation, but at the same time minimising the potential adverse effects that may be associated with chronic treatment. The focus of future oncology trials will require careful optimisation of biomarker-coupled combination approaches with agents that synergistically target multiple aberrant pathways in PDAC.
